# Modeling the Pre-Industrial Roots of Modern Super-Exponential Population Growth

**DOI:** 10.1371/journal.pone.0105291

**Published:** 2014-08-20

**Authors:** Aaron Jonas Stutz

**Affiliations:** 1 Division of History & Social Sciences, Oxford College of Emory University, Oxford, Georgia, United States of America; 2 Department of Anthropology, Emory University, Atlanta, Georgia, United States of America; University College London, United Kingdom

## Abstract

To Malthus, rapid human population growth—so evident in 18th Century Europe—was obviously unsustainable. In his *Essay on the Principle of Population*, Malthus cogently argued that environmental and socioeconomic constraints on population rise were inevitable. Yet, he penned his essay on the eve of the global census size reaching one billion, as nearly two centuries of super-exponential increase were taking off. Introducing a novel extension of J. E. Cohen's hallmark coupled difference equation model of human population dynamics and carrying capacity, this article examines just how elastic population growth limits may be in response to demographic change. The revised model involves a simple formalization of how consumption costs influence carrying capacity elasticity over time. Recognizing that complex social resource-extraction networks support ongoing consumption-based investment in family formation and intergenerational resource transfers, it is important to consider how consumption has impacted the human environment and demography—especially as global population has become very large. Sensitivity analysis of the consumption-cost model's fit to historical population estimates, modern census data, and 21^st^ Century demographic projections supports a critical conclusion. The recent population explosion was systemically determined by long-term, distinctly pre-industrial cultural evolution. It is suggested that modern globalizing transitions in technology, susceptibility to infectious disease, information flows and accumulation, and economic complexity were endogenous products of much earlier biocultural evolution of family formation's embeddedness in larger, hierarchically self-organizing cultural systems, which could potentially support high population elasticity of carrying capacity. Modern super-exponential population growth cannot be considered separately from long-term change in the multi-scalar political economy that connects family formation and intergenerational resource transfers to wider institutions and social networks.

## Introduction

**Figure 1:**
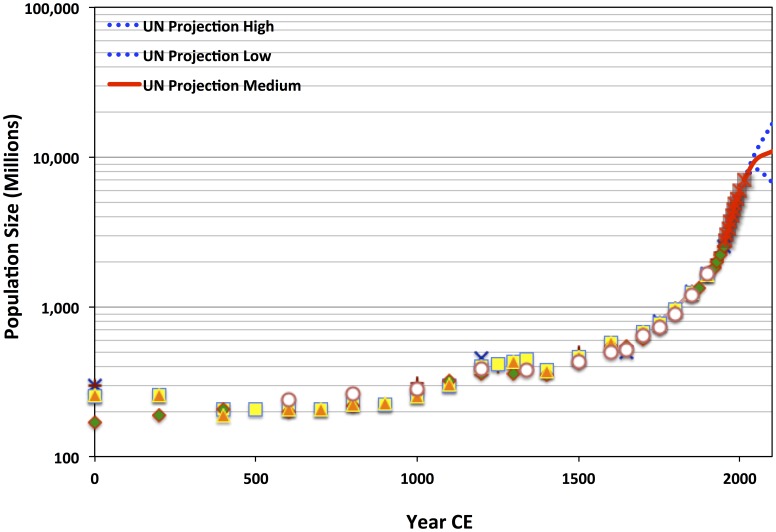
Semilog plot of historical estimates from 1–1950 CE (various shapes), recent UN census data for 1955–2012 (red asterisks with gray background), and 21^st^ Century projections based on variation in fertility and mortality rate trends (solid red line bounded by upper and lower range blue dotted lines), showing a logistic growth pattern with a remarkably rapid acceleration during the 19^th^ and 20^th^ centuries. Although uncertainty in the 21^st^ Century UN population projects encompasses continued growth to ca. 17 billion, as well as imminent decline toward ca. 6–7 billion, it is clear that demographic growth began decelerating over the past 20 years, and that deceleration is continuing. Data from refs. [4], [23], [24].

Malthus published the first edition of his essay on limits to human population in 1798 [1]. Since then, diverse stresses—caused by political violence and marginalization, poverty, poor nutrition, and infectious diseases—seem not to have checked global growth in the least [2], [3]. Only a widening decline in fertility has recently begun to slow global demographic growth (Fig. 1) [4]. Humanity's modern population rise has profoundly impacted and transformed ecosystems around the world [5]–[9]. The modern human population explosion co-occurred historically with what has variously been described as the modern technological, economic, human capital, and ideological eras [10]–[17]. It also co-occurred with the demographic and epidemiological transitions to lower mortality, initially involving geographically patchy variation in fertility, followed by recurrent, broadening birthrate declines [2], [18], [19]. The dynamics of the ongoing, globalizing demographic transition are usually modeled and discussed—at least implicitly—as if they were distinct, driven by qualitatively different factors than those shaping pre-industrial population dynamics [12], [20], [21]. Put in Malthusian terms, did the cultural, political, and economic dimensions of modernity allow human populations suddenly and temporarily to escape earlier environmental constraints, which had apparently remained in place well into the 18^th^ Century? Of course, Malthus would skeptically expect the answer to this latter question to be “no”. Nonetheless, in seeing only indications that 19^th^ Century population would face insurmountable limitations to growth, Malthus would surely be surprised that global population has roughly doubled three times since 1798 (see Fig. 1).

Cohen's landmark—and remarkably simple—model of coupled dynamic change in population and ecological carrying capacity supports the plausible claim that the cultural and environmental transformations underlying industrialization and modern transportation and communication technologies had systemic roots stretching many centuries prior to Malthus's initial publication of his *Essay* [22], [23]. Cohen's model provides an elegant exploratory analytical tool for investigating recent global human population change. In this article it is argued that an extension of Cohen's original coupled-difference-equation model facilitates analytically evaluating two alternative hypotheses: (1) that the demographic transition was caused by an abrupt systemic transition—perhaps driven by a cascading socio-cultural rupture—in the late 18^th^ or early 19^th^ Century, or (2) that more ancient, preindustrial initial conditions determined long-term patterns in the elasticity of carrying capacity. In order to consider these possibilities, this study carries out a sensitivity analysis of the original Cohen (OC) model and the extended “consumption-cost” (CC) model. The aim is to compare modeled population trajectories—deterministically shaped by interaction with a social-scale-dependent carrying capacity, which is elastic in relation to change in population—with demographic estimates that are independently based on historical data concerning trends in fertility, mortality, land area occupied, and population densities (Table 1; see Fig. 1) [23], [24]. The new—and also very simple—modification of Cohen's original model can help to clarify the limits to positive elasticity in carrying capacity relative to human population.

**Table 1:** Global human population (millions of people), 1–2012 CE.^1^

Year CE	McEvedy & Jones	Kremer	Biraben	Blaxter	Clark	Haub	UN 1999	Average (1750–2012)
1	170	170	252	255	256	300	300	
200	190	190	257	256				
400	190	190	206	206	254			
500	190		207					
600	200	200	208	206	237			
700	210		206	207				
800	220	220	224	224	261			
900	240		222	226				
1000	265	265	253	254	280		310	
1100	320	320	299	301				
1200	360	360	400	400	384	450		
1250			417				400	
1300	360	360	431	432				
1340			442		378			
1400	350	350	375	374				
1500	425	425	461	460	427		500	
1600	545	545	578	579	498			
1650	545	545			516	500		
1700	610	610	680	679	641			
1750	720	720	771	770	731	795	790	757
1800	900	900	954	954	890		980	925
1850	1200	1200	1241	1241	1190	1265	1260	1227
1875	1325	1325						1325
1900	1625	1625	1634	1633	1668	1656	1650	1643
1920		1813					1860	1837
1930		1987					2070	2029
1940		2213					2300	2257
1950	2500	2516	2530	2513		2516	2520	2516
*1955*							*2752*	*2752*
*1960*							*3020*	*3020*
*1965*							*3336*	*3336*
*1970*							*3698*	*3698*
*1975*							*4079*	*4079*
*1980*							*4448*	*4448*
*1985*							*4851*	*4851*
*1990*							*5292*	*5292*
*1999*							*6000*	*6000*
*2012*							*7000*	*7000*

1 Historical estimates for 1–1950 CE are from refs. [72]–[78]. The UN global census data for 1955–2012 is from ref. [24], which provides an open-access web-based summary of these data. The historical world population estimates are also summarized by Cohen [23] in his Appendix 2. Note that the average population values—which are used to calculate 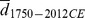 (the distance for a given model population trajectory from the average population estimate/census value for the 1750–2012 data)—exclude duplicate estimates, in which a later study relies on an earlier study's result (e.g., Kremer's extensive use of the earlier estimates from McEvedy & Jones [74], [77]).

## Background

Cohen's seminal formalization of human demographic growth defines *scale-dependent social organization of resource extraction and processing* as a factor that dynamically couples population change with changes in environmental constraints on population increase. In the OC model Cohen modifies the classic Verhulst-Pearl logistic growth equation to define environmental carrying capacity as a historically dependent variable—that is, a function 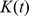, so that 
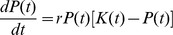
 [22], [23]. He accounts for change in 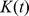, beginning with the basic assumption that carrying capacity is elastic with respect to population size, so that its history-dependent dynamic would follow a rate of change proportional to that of change in the population, 
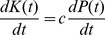
. In grappling with the carrying capacity concept, Cohen is taking on a theoretically implied phenomenon so general that—all too often—its formalized abstraction obscures, rather than heuristically clarifies, the systemic ecological factors shaping population growth patterns [25]. What is new is that Cohen concisely models endogenously driven logistic population change, whose trajectory may be usefully compared with standard Verhulst-Pearl trajectories [26]. Basically, Cohen establishes a standard of comparison for evaluating whether human population growth is positively shaped by social-network, rather than constrained by exogenous, fixed, niche-defined limits. It is especially theoretically relevant for studying human biocultural evolution that—in defining carrying capacity as elastically responding to population change [22], [23]—Cohen elegantly supports a dynamic niche construction approach [27] to studying our socially intensive, transfer-dependent life-history adaptations.

**Figure 2:**
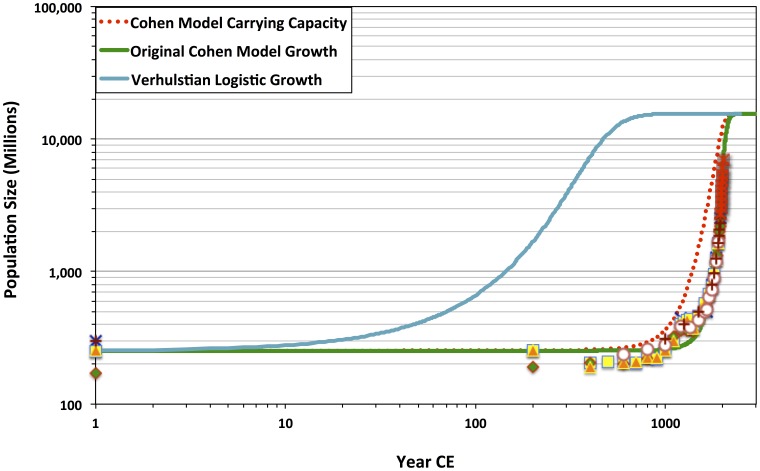
Logarithmic plot comparison of population-trajectory fit between the standard Verhulst logistic growth model and Cohen's discrete-step coupled difference equation model of human population and carrying capacity growth. The original Cohen model shows the population trajectory based on the parameter values and initial population and carrying capacity conditions as in ref. [22]. The Verhulst trajectory is based on the same population initial condition, 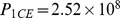, as for the Cohen model; static carrying capacity, *K*, is set to the Cohen model asymptotic equilibrium value 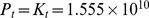. The natural intrinsic rate of growth, *r*, is conservatively set to 0.01, well below estimates of human *r_max_* [26]. Historical population estimate and UN census data shown are as in Fig. 1.

Indeed, he further suggests that the coefficient *c* (see above) may be usefully defined in relation to 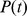. Culturally structured values, preferences, beliefs, and interlinked—often competing—institutions are initial conditions that set a (theoretically) constant threshold size, 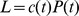, at which an additional brain or pair of hands no longer offers any economy of scale. Thus, with 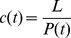, carrying capacity change decelerates when 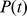 surpasses 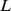. Using discrete difference equation forms, the OC model illustrates how a coupled system of change in population and carrying capacity yields a surprisingly close centennial and millennial-scale fit with historical population estimates and modern population data (Fig. 2).

Indeed, with starting conditions set at 1 CE, the OC model seemed—at first glance—to account for the super-exponential growth that has occurred over roughly the past 200 years. Although the world has absorbed a net gain of roughly 1.5 billion people since the Cohen equations were published, the OC model further presciently supported current United Nations projections that global growth rates will actually level off and approach zero—or possibly even decline—in this century [4].

### Modeling Resource-Transfer Impacts on Carrying Capacity

Despite the explanatory promise of the OC model, it has not been subjected to thorough theoretical and analytical scrutiny. One key limitation in the OC model actually involves an admitted, explicit assumption [22], [23]: resource-extraction efficiency and costs associated with investment in fertility and intergenerational resource transfers simply attenuate as 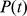 approaches and then surpasses 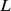. In the OC system dynamics, as 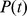 increases, change in carrying capacity approaches zero, reaching a demographically stationary, Malthusian steady state. Consequently, once it is gained, carrying capacity cannot be lost in the OC model.

In order to address this issue, the CC model involves a simple redefinition of the variable 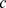
—that is, the coefficient of relationship between change in carrying capacity and change in population, or the *population elasticity of carrying capacity*:
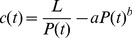
(1)


The coefficient 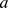 is a constant rate of discount on marginal non-linear ecological impacts of consumption—including biotic and abiotic resource depletion—involved in family formation, investment in somatic maintenance, fertility, and transfers to offspring and descendants. Relevant values of 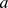 are constrained so that 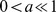, reflecting the expectation that consumption costs will impact carrying capacity growth only at larger population sizes. The non-linear impacts themselves are modeled by power coefficient 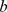. As aggregate resource consumption rates increase, resources may get depleted in disproportionately positive relationship to population. Values of 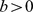 should yield realistic results. With the redefinition of 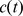, the differential equation for change in carrying capacity becomes: 
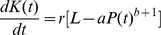
. Consumption costs are expected to rise non-linearly because more than individual food and water needs are required to sustain high numbers. Also needed—or demanded—are material resources for extraction, processing, transportation, distribution, and even material support for family formation, intergenerational transfer and consumption practices. Moreover, at large population sizes factional and institutional conflicts emerge over land-use for food production, transportation and storage; potable water extraction, transportation and storage; and non-food and water resource extraction, transportation, consumption, and discard. Thus, at a certain high-population threshold, each additional person has a larger and larger negative impact on resource acquisition and distribution, raising the costs for survival, fertility, and intergenerational transfers, while leading in some cases to overexploitation of renewable resources and depletion of finite ones.

### The Cost of Reproductive Success in Society

A second, important area left unexplored is how the OC model is defined relative to the human natural intrinsic rate of increase, 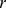. Cohen presents his original model for heuristic—rather than analytical—purposes [22]. However, the value of 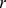 that he uses for illustrative reasons scales to an infinitesimal per capita annual reproductive rate of roughly 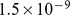 offspring. The life-history strategy of *Homo sapiens*—with distinctively long maternal gestation and juvenile growth periods; typically long great-ape lactation periods; and a variable age at last reproduction between 35–50 years for both sexes [28]–[30]—does limit *r_max_* in a population with stable age structure with a roughly equal sex ratio. Yet, this natural intrinsic rate of increase may be estimated to be roughly 10^−2^<*r_max_*<10^−1^ (measured as a continuous rate of offspring production per capita per annum) [26], [31]–[33]. This is nearly seven orders of magnitude greater than the *r*-value yielding good OC-model fit to independent population estimates. A surprising implication of Cohen's emphasis on endogenous cultural system growth in carrying capacity is that “*r*” takes on a new, virtually flipped definition. Cohen's revision of the classic Verhulst-Pearl logistic growth function swaps “driver” and “destination”. The basic Verhulstian (and Malthusian and Darwinian) expectation is that a high natural intrinsic rate of increase is limited by logistic growth to an ecological limit determined exogenously to the population itself [34]. Thus, natural increase drives growth, and essentially constant exogenous ecological limits determine the steady state level. However, in Cohen's formulation, substantial population growth only occurs when endogenous cultural niche construction processes—possibly in combination with exogenous changes, such as climatic amelioration—raise carrying capacity, 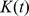, sufficiently above prevailing population, 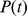. Moreover, this can only occur through prior cultural evolution of the *potential* for economies of scale, *L*.

**Figure 3:**
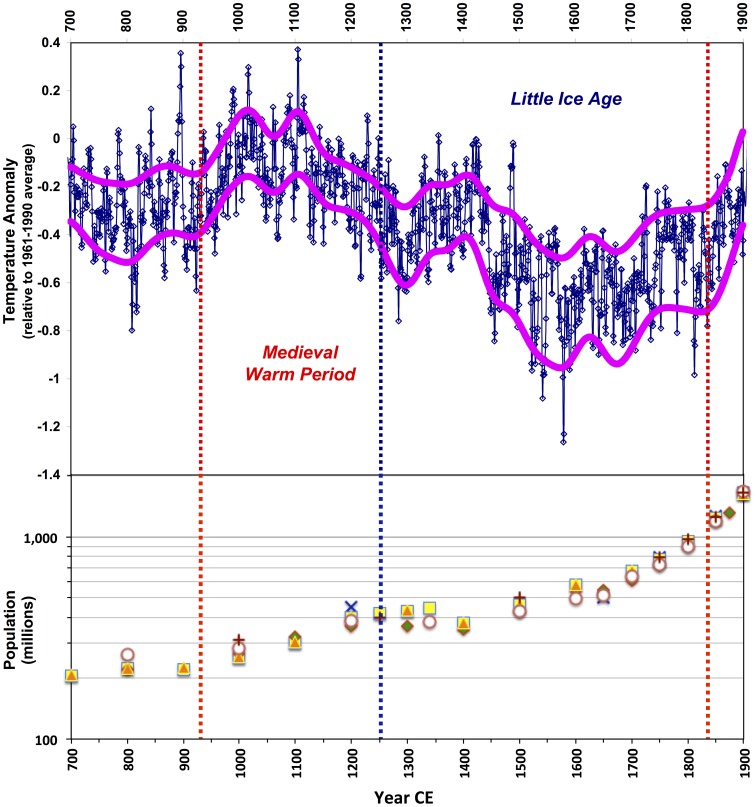
Northern Hemisphere temperature variation, 700 AD – 1900 AD, juxtaposed with historical population estimates. Historical climate proxy data are from the supplementary materials in ref. [35]. Historical population data as in Fig. 1.

In fact, the independent historical estimates suggest that, following a long period of demographic stasis, global population nearly doubled between ca. 900–1300 CE—an interval that closely coincides with the brief climatic interstadial known as the Medieval Warm Period [35]–[37] (Fig. 3). Cohen's model supports a more plausible explanation of how marginal increases in ecological productivity and reductions in temperature and precipitation extremes supported a disproportionate, supra-marginal expansion in human numbers, at such a broad geographic scale. The prior, independent political-economic emergence of potential for economies of scale, *L*, in different parts of the world—across Eurasia, in Africa, the Pacific Islands, and the Americas [38]—would have allowed uninterrupted cumulative growth in carrying capacity, 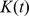, which exhibited a positively elastic response to Medieval Warm Period climatic amelioration.

In Cohen's framework, the intrinsic rate of increase, *r*, is implicitly the small amount of population growth that can be achieved when prevailing cultural systems limit the social mobilization of family formation, resource extraction, and consumption to near-replacement levels, so that 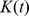
is only slightly larger than 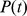. We may distinguish this cultural, social-network-limited rate of increase, *r_soc_*, from the natural maximum intrinsic rate of increase, *r_max_*, so that:
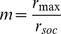
(2)


In general, the human case involves 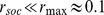. Now, Cohen labeled the parameter *c(t)* [see equation (1) above] the “Condorcet parameter”. The formalization of change in carrying capacity as exhibiting positive elasticity with respect to change in population is a nod to Condorcet's optimistic notion that human ingenuity would inevitably solve problems posed by growing populations [22]. Although Cohen did not explicitly recognize or derive the substantial change in *r*'s definition that he had wrought, the relationship term, *m*, between *r_max_* and the much smaller *r_soc_* may be dubbed the “Marx parameter”. As Karl Marx stated in *Grundrisse*, “The human being is in the most literal sense a *zoon politikon*—not merely a gregarious animal, but an animal which can individuate itself only in the midst of society… Whenever we speak of production, then, what is meant is always production at a definite stage of social development—production by social individuals” [39]. Marx emphasized the potential of social relations of production to define and isolate the individual as an Aristotelian “political animal”, but the political-economically mediated, dramatic dilution of *r_max_*—a phenomenon preliminarily suggested by Cohen's initial work with the OC model—brings into focus the profoundly *non-individuating* systemic tie between self and society. Biocultural evolution may be substantially defined by the especially complex interconnections in which the individual is linked by the family and the wider social networks through which family is defined culturally—in the process of family members working to obtain, hold, and consume resources, in order to invest in fertility and transfer resources to multi-generational sets of descendants.

**Figure 4:**
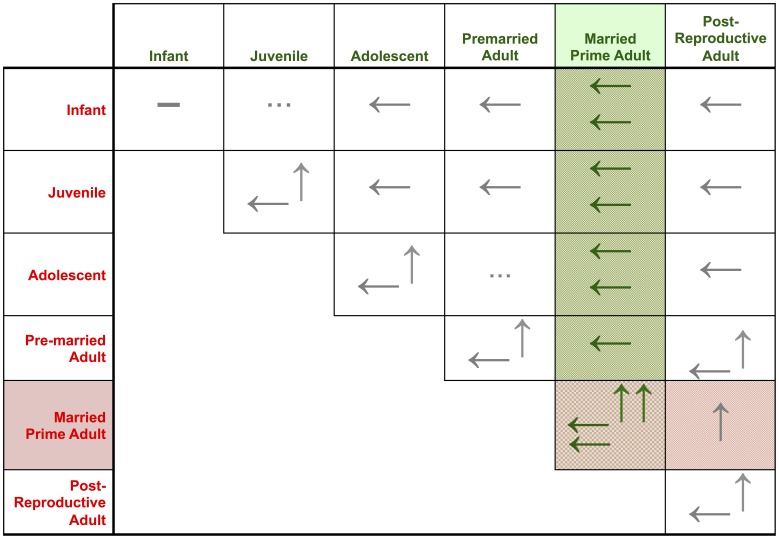
Typical preindustrial flow of transfers in human groups among all life history stages, emphasizing the importance of giving and receiving for prime adults engaged in family formation, investment in fertility, and offspring and descendant care. Transferred resources include consumable calories, material capital, social capital, technological know-how and environmental knowledge, and cultural competence. The preponderance of exchange within life history stages occurs among married adults and involves a combination of material and social capital, including reputation. In turn, married adults account for the bulk of transfers to other life stages. Modified after ref. [37].

Long-term systemic change in the functional dilution of *r_max_*, then, is arguably an important, yet largely unrecognized property of socio-politically complex human cultural systems. As Graeber has recently noted—in discussing the practical, cosmological, and ideological dimensions of kingship, state violence, ritual violence, and social order in pre-industrial, non-literate contexts—the definition and management of reproductively potent human populations as resources to control, sustain, or exploit has likely always been a well-focused political concern for factional and individual interests in culturally structured social networks [40]. In any demographically sustainable human population, individuals will face a continuous cost for acquiring and holding resources for family formation, investing in fertility, and providing transfers to dependent offspring and related descendants. This simply reflects the intensity of resource transfers embedded in human life history adaptations [41]–[48], set in large social networks with self-organizing spatio-temporally and functionally hierarchical structures [49]–[53] (Fig. 4). In using his model to interpolate the population trajectory between 1 and 1995 CE—yielding a visually satisfying fit to intermediate historical estimates and modern census data—Cohen implicitly supports the expectation that *m* does not vary across network scales, at least above a certain threshold in which socio-politically and economically complex cultural systems are constituted by geographically widespread metapopulations, with total census sizes greater than roughly 10^6^ or 10^7^.

In the OC and CC models alike, the Marx parameter measures the individual's cost of biological reproduction in society, relative to investment needed to raise aggregate carrying capacity marginally. Both models are specified according to plausible general premises about how population change can drive niche construction, recursively altering resource availability in a way that may cause an elastic, disproportionately positive or negative response. Carrying capacity elasticity, *c(t)*, is simultaneously a coefficient of population change, 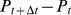, and recursively determined by *P_t_*; consequently, carrying capacity changes non-linearly over time, and the long-term coupled dynamics of *P_t_* and *K_t_* are highly sensitive to initial conditions. This logically raises the possibility that—in the context of the historical, roughly scale-invariant Marx parameter definining *r_soc_* relative to *r_max_*—late prehistoric and early historic biocultural evolutionary changes influencing the potential for economies of scale, *L*, could have first delayed impacts on carrying capacity and population, only to give way to modern accelerating, super-exponential growth.

## Methods

Multiple historical estimates and recent United Nations census data provide a widely accepted reconstruction of long-term global population change. These data and references to the original sources are openly available on the United States Census Bureau website [24] and are shown in Table 1 and on Figs. 1 and 2. This section describes a method of sensitivity analysis of the fit of OC and CC model trajectories to the independently estimated historical data and modern census values. All calculations and analyses based on the methods were carried out in Microsoft Excel.

### Coupled Difference Equations

As Cohen points out [22], [23], it is useful to model coupled population and carrying capacity change with difference equations, in order to explore complex dynamics across annual or generational intervals. Cohen writes the discrete-step difference equation for logistic growth:
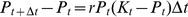
(3)


In constructing the discrete-step difference equation for carrying-capacity change, we substitute 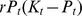 for 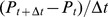, so that 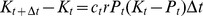. Following the definition of *c(t)* in equation (1), we write:
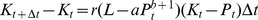
(4)


From the equivalence 
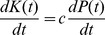
, the population elasticity of carrying capacity—that is, the responsiveness of carrying capacity, 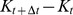, to a given change in population, 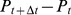
—is simply *c_t_* [see equation (1)].

Population trajectories following the CC model may be generated from initial conditions by coupling equations (3) and (4). The OC model may be generated from initial conditions by calculating values of carrying capacity, population, and change in those variables by setting parameter *a* = 0, and coupling equation (3) with the CC model's equation (4).

### Sensitivity Analysis

The historical estimates for global population in the year 1 CE range from 170 to 300 million. The United Nations and the United States Census Bureau use country-specific census counts and data on mortality and fertility rates to calculate that world population reached 7 billion in 2012. Sensitivity analysis can evaluate how well the OC and CC models interpolate global population trajectories between 1 and 2012 CE, in terms of their fit to independent census measurements within the 1–2012 CE interval. It can also measure the fit of the projected population in 2100 CE to independently derived United Nations projections, which use trends in national rates of mortality and fertility. In order to trace how the OC and CC models behave, the sensitivity analysis considers six cases defined by a low and high *P_1 CE_* value (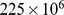 and 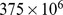), respectively, each combined with three separate *r_soc_* values (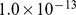, 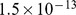, and 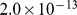). For each trajectory, *K_t_*, *P_t_*, and *c_t_* were calculated for annual intervals—from initial conditions at 1 CE to 2500 CE—recursively using equations (3) and (4). General fit of the resulting modeled time series was measured as the average distance, 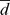, from the historical global population estimates and census data for the years 1750 to 2012 CE. Here, 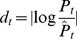. For the estimated global population intervals from 1750–1950 CE, 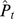 is the arithmetic average of all measurements for a given year. For the UN population values from 1955–2012, 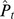 is simply the mid-year global census estimate. The year 1750 CE was chosen as the beginning of the measurement of fit between modeled *P_t_* values and independently estimated 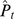 values, because national censuses began to be made in a widening number of countries around that time [2], [23]. Thus, the period from 1750 to the present includes increasingly precise and accurate population measurements.

**Figure 5:**
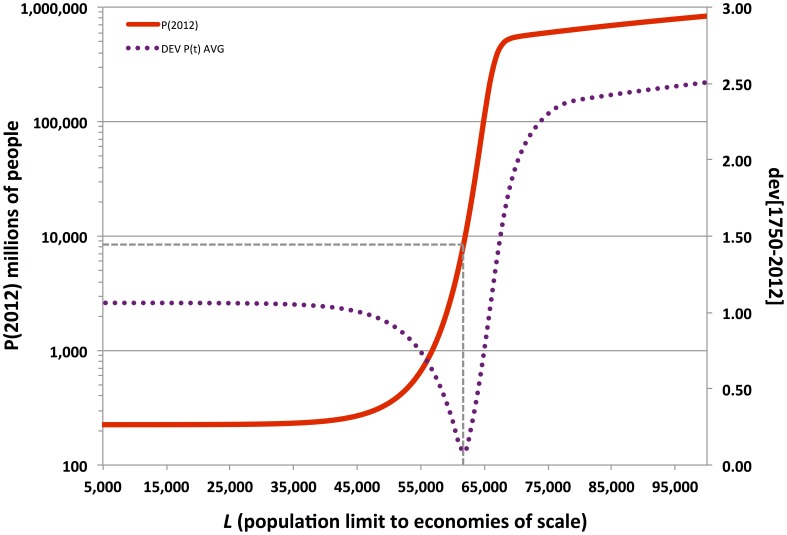
Scatterplot of modeled population in 2012 CE (*P_2012 CE_*) and the overall modeled trajectory's deviation from historical estimates and census data (here labeled *dev[1750*–*2012]* for clarity, defined as 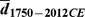 in the text and Tables 2 and 3) versus the cultural systemic limit to achieving economies of scale, *L*. The case shown involves 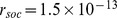; 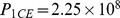; and 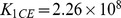.

Preliminary analysis determined that values of 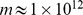 (yielding values of 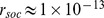) generate population trajectories more closely concordant with the historical estimates and census data than the smaller value (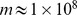) used by Cohen. In fact, the OC model trajectory shown in Fig. 2 yields a distance value 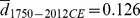; in comparison, best-fit trajectories based on the higher Marx parameter reduce 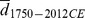 by a factor of roughly three or four. In addition, values of *L*—the limit to economies of scale—that are larger than Cohen's illustrative value of 3.7 billion also appear to offer better-fit trajectories. Holding *m* constant, the complete annually resolved OC trajectories were calculated for values of *L* from 5–200 billion. For each OC trajectory calculated in each “*L*-scenario”, the 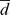-value was recorded, along with *P_1800 CE_*, *P_2012 CE_*, *P_2100 CE_*, and *P_2500 CE_*. Fig. 5 demonstrates how the value of *L* that minimizes 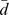 is sensitively dependent the scenario's initial conditions in the OC model. The OC trajectory that minimized 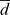 for each case provided the value of *L* used to evaluate the CC model's fit to the historical estimates and census data. Holding *L* and *r_soc_* constant, combinations of *a* and *b* values were used to generate new population time series. The parameter *a* was varied between 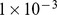 and 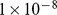 by whole orders of magnitude. For each level of *a*, the parameter *b* was varied between 0.1 and 2.5 in increments of 0.1. Finally, the value of *b* that contributed to minimizing 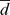 in the coarse analysis was used as the center of a finer examination of best fit across values of *a* between 0.1 and 2.5. Again, for each CC model trajectory, the 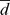-value was recorded, along with *P_1800 CE_*, *P_2012 CE_*, *P_2100 CE_*, and *P_2500 CE_*.

## Results

Tables 2 and 3 summarize the variation in the OC and CC models' fit to historical and census data, respectively. In each of the six OC cases examined, there is a value of *L* that markedly minimizes deviations from the recent historical estimates and census data for the interval 1750–2012 CE. From this perspective, the OC model does indeed provide good fit with historical population data, closely tracking modern super-exponential growth over the past two centuries. However, these best-fit OC population trajectories involve limits to economies of scale, *L*, on the order of 10's of billions of people. Although the best-fit OC model successfully interpolates the population trajectory from the 1 CE mean estimate (225 million people) to the 2012 CE global human numbers, it no longer predicts the slowdown in population growth that has been confirmed by the past two decades' census and demographic rate data (see Fig. 1).

**Table 2:** Sensitivity analysis results for the Original Cohen (OC) model.

*P*_1 CE_	*r*_soc_	*OC Model (no consumption costs for growth in carrying capacity)*				
		*L*	*d_avg_*_(1750_–_2012 CE)_ 1	*P*_1800 CE_	*P*_2012 CE_	*P*_2100 CE_
2.25×10^8^	1.00×10^−13^	6.1677×10^10^	0.048	6.1026×10^8^	8.1964×10^9^	7.8536×10^10^
	1.50×10^−13^	3.9684×10^10^	0.044	6.3341×10^8^	7.7096×10^9^	5.8960×10^10^
	2.00×10^−13^	2.9045×10^10^	0.042	6.5758×10^8^	7.6053×10^9^	4.9666×10^10^
3.75×10^8^	1.00×10^−13^	6.0711×10^10^	0.030	8.6160×10^8^	6.9992×10^9^	4.4469×10^10^
	1.50×10^−13^	3.9112×10^10^	0.033	8.9298×10^8^	6.7589×10^9^	3.6172×10^10^
	2.00×10^−13^	2.8640×10^10^	0.035	9.1781×10^8^	6.5800×10^9^	3.0971×10^10^

1 The value in this column is the average distance, *d_t_*—as defined in the text—between the OC model population size and the estimated or measured population size for year t from 1750 to 2012. Only the estimated or measured years, shown in Table 1, were included in the calculation of 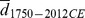
_._

**Table 3:** Sensitivity analysis results for the extended consumption cost (CC) model.

*P*_1 CE_	*r*_soc_	*CC Model (consumption costs drag on growth in carrying capacity)*						
		*a*	*b*	*d_avg_*_(1750_–_2012 CE)_ 1	*P*_1800 CE_	*P*_2012 CE_	*P*_2100 CE_ 2	*% error P*_2012 CE_ 3
2.25×10^8^	1.00×10^−13^	5.0×10^−13^	1.92	0.047	6.0980×10^8^	7.5609×10^9^	1.4505×10^10^	8.01
	1.50×10^−13^	5.0×10^−14^	2.10	0.044	6.3321×10^8^	7.3432×10^9^	1.5301×10^10^	4.90
	2.00×10^−13^	5.0×10^−14^	2.04	0.043	6.5736×10^8^	7.3043×10^9^	1.6335×10^10^	4.35
3.75×10^8^	1.00×10^−13^	2.0×10^−14^	2.20	0.031	8.6111×10^8^	6.7968×10^9^	1.6684×10^10^	−2.90
	1.50×10^−13^	8.0×10^−14^	2.00	0.035	8.9200×10^8^	6.5294×10^9^	1.6364×10^10^	−6.72
	2.00×10^−13^	2.0×10^−14^	2.10	0.037	9.1717×10^8^	6.4193×10^9^	1.6579×10^10^	−8.30

1 The values in this column are calculated as in Table 2.

2 The values of *P_2100 CE_* yielded by all six sensitivity cases examined for the CC model fall within the upper range of UN demographic projections for global human census size in the year 2100 [4].

3 The values in this column are the % deviation of the CC model value of *P_2012 CE_* from the observed value of 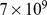.

**Figure 6:**
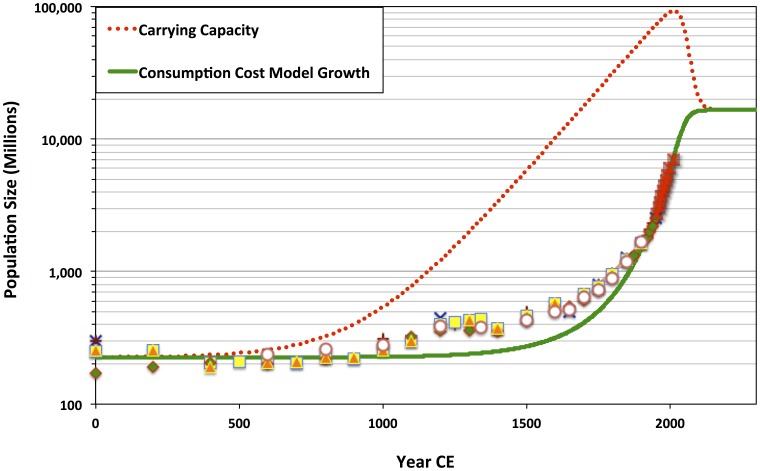
Consumption-cost (CC) model trajectory for the case involving 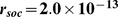; 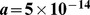; 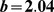; 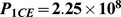; and 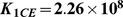. See Table 3 for additional details.

In fact, the historically best-fit trajectories of the OC model project global super-exponential growth to continue unabated through the 21^st^ century. For example, the OC model with parameters set as in Fig. 6 yields a population in 2100 CE at 59 billion. The Malthusian steady state is reached by 2500 CE, at 286 billion people. When carrying capacity, *K_t_*, can grow for so long without resource depletion or other systemic constraints, the velocity of population only slows down after two or three doubling periods past *L*. Only at this stage do population growth and carrying capacity growth converge asymptotically toward zero. Needless to say, 286 billion exceeds any of the historical and modern estimates of global human population saturation that Cohen reviewed [23].

**Figure 7:**
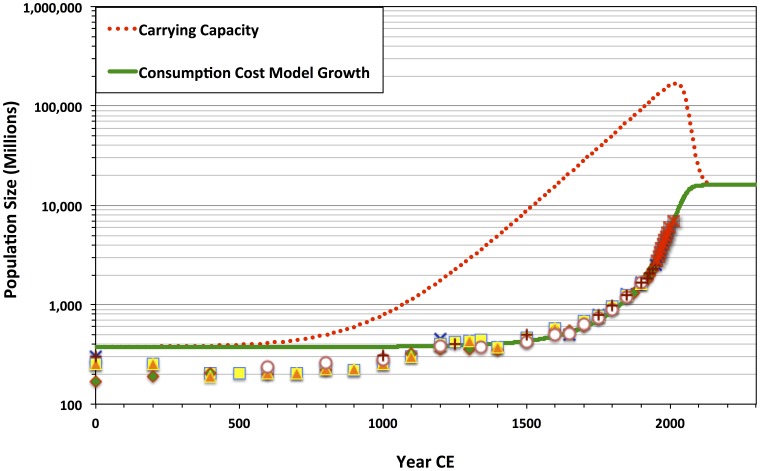
Consumption-cost (CC) model trajectory tuned by inspection to fit *P_2012 CE_* = 6.999 billion. In this case, 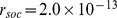; 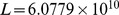; 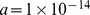; b = 2.54; 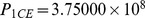; and 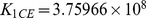. This case results in 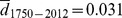 and 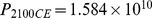, demonstrating that—according to the CC model—initial conditions established between 1 and 1500 CE can largely account for the historical pattern of modern super-exponential growth and the projected trend of population deceleration through 2100 CE.

In contrast, although the fit of the CC model to historical estimates and census data is generally similar to that of the OC model, it further achieves remarkable agreement with independent demographic projections of declining population growth through the 21^st^ Century. Fig. 7 illustrates how finer variation in parameter values and initial population and carrying capacity conditions may be tuned to generate closer agreement between the CC model trajectory and the 21^st^ Century UN demographic projections.

## Discussion: The Costs of Consumption in Human Population Systems

The sensitivity analysis of the original Cohen model and the consumption-cost extended version supports the hypothesis that the super-exponential population growth of the 19^th^ and 20^th^ centuries was only proximately caused by modernity's organizational, ideological, and technological changes. Although only one line of evidence, the sensitivity analysis of the OC and CC models suggests that recent population growth did not involve a simple and sudden, technology-, ideology-, or human capital-dependent transition from Malthusian constraints. Those constraints would have remained. However, during the period of rapid global population growth, the marginal gains in carrying capacity, *K_t_*, yielded by economies of scale continued to exceed the costs of adding more mouths to feed (see Figs. 6 & 7).

This result potentially illuminates why Malthus did not anticipate the modern human population explosion. Malthus incorrectly assumed that food supply was mainly dependent on area under cultivation. 19th and 20th Century aggregate food-supply increase outstripped population growth, not only due to rising agricultural yields, but also due to organizational, legal, transportation, processing, storage, educational, and ideological innovations—all of which were network-scale dependent and mutually interdependent [54]. Recent theoretical treatments of modern demographic dynamics emphasize how material and energy-intensive intergenerational resource-transfer strategies have simultaneously favored rapid fertility declines and rising consumption rates [10]–[12], [55], [56], but the OC and CC models alike suggest that modernity's most marked biocultural trade-off—reallocating resources from investment in fertility to per capita transfers—has not significantly increased global aggregate potential for achieving economies of scale. Rather, substantially earlier biocultural evolutionary developments—likely involving previously unrecognized systemic constraints on how family formation practices and intergenerational transfer norms structure and are structured by wider economic coordination and competition over extraction, production, and distribution—would have set limits to growth in the potential for achieving economies of scale, represented in the OC and CC models by *L*.

It must be remembered that these models, as presented, involve explicit simplifying assumptions about both the potential for economies of scale, *L*, and the intrinsic social-network-mediated rate of increase, *r_soc_* (mainly shaped by the Marx parameter, *m*). These factors are hypothesized to be essentially constant features of historical, socio-politically complex, geographically widely interconnected cultural systems and the populations that constitute them. The general coupled difference equation approach to population and carrying capacity change may prove helpful for studying regional—rather than supra-continental or global—demographic dynamics. Here, though, more detailed parameterized models will likely yield greater insight into demographic history variation among such relatively local contexts [57]–[61]. In this study the OC and CC models are scientifically relevant because they evoke plausible supra-regional contexts in which the parameters *L* and *r_soc_* are roughly constant. Here, it becomes clear that the OC and CC models are scientifically useful only insofar as they help us think through and refine relevant hypotheses about very complex interactions among human populations, the social networks they constitute, and the environments with which they extract, transform, and exchange matter and energy. From this perspective, inspection of the results for the CC model suggests that we may be able to trace possible cultural evolutionary transitions in levels of *L*, *r_soc_*, and *K(t)*. For example, comparison of the six cases analyzed in Tables 2 and 3 shows that—for the OC and CC models alike—the higher value of *P_1 CE_* (375 million) yields the better fit to historical population estimates. This initial population value is also consistent with a more complex demographic history, in which population growth from the first millennium CE through the Medieval Warm Period (see Fig. 3) may have involved a substantial increase in the ideological, institutional, and technological foundations for potential economies of scale, *L*. Although the Marx parameter, *m*—that is, the average cost of forming a family, investing in fertility, and obtaining and holding transfers to offspring—would have remained very high compared to *r_max_*, it may also have declined significantly during the apparent Medieval Warm Period era of demographic growth.

In all instances, key political-economic changes—involving organizational, ideological, and technological innovations—would have shaped the potential for economies of scale, supporting very high population elasticity of carrying capacity around ca. 1500 CE. Indeed, in the closely fit CC model trajectories shown in Figs. 6 & 7, carrying capacity, *K_t_*, begins increasing faster than population, *P_t_*, around 1500 CE. Yet, the extraordinary fit of the CC model trajectory to historical estimates, census data, and 21^st^ Century demographic projections suggests that critical political-economic developments were in place much earlier. Whatever the exact nature of these prior changes in organization, ideology, and technology, they were initially adopted in biocultural evolutionary environments in which population growth was limited over large continental or supra-continental land areas (that is, >ca. 10^6^–10^8^ km^2^), more often than not subject to growth-limiting Malthusian constraints over generational to centennial timescales. The CC model is highly sensitive to initial conditions, and the best-fit trajectories all involved initial carrying-capacity levels (*K_0_*) only slightly greater than actual initial population levels (*P_0_*). In other words, it appears that even with beginning conditions incorporating very high limits on economies of scale, *L*, those same initial conditions would have also had the global human population just barely under the prevailing carrying capacity—that is, very close to immediate Malthusian limits on growth, 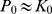. This would establish long-term suppression of population rise, while contributing to a nonlinear, positively elastic carrying-capacity response. Significant cultural structures determining population-dependent limits on achieving economies of scale would have emerged when continental-scale populations were largely stationary over a period of many generations or centuries. The historical estimates suggest that global population growth was stagnant during the entire first millennium CE, although archaeological and historical evidence documents comparably dynamic variation in migration and settlement patterns, on the one hand, and political economy, on the other [38], [62]. Here, the OC and CC models help to direct our attention toward a new hypothesis. Developments in cultural system complexity—emerging in different parts of the world in the centuries and millennia prior to 1500 CE—were more systemically important in setting the stage for the recent population explosion than was any specific modern technology or ideology introduced during or after the 18^th^ Century.

How then might *unambiguously pre-industrial and pre-modern* biocultural evolutionary processes have generated an enormous potential systemic capacity for achieving what turned out to be 19^th^ and 20^th^ Century industrial and information economies of scale? The answer arguably lies in dynamics of competition, power, and inequality [10], [38], [63], [64]. Let us assume a prehistoric set of initial conditions—emerging during the Holocene, between the origins of agriculture and the establishment of states and urban settlement systems—in which substantial within-population heterogeneity in political power and biological well-being have become institutionalized, as continental-scale meta-populations approached a stationary steady-state. Under such conditions, Malthusian regulation would have largely prevailed. However, within populations an elite segment would have been able to exploit differential access to material resources, information, and media of communication, in order to mobilize labor on risky or expensive organizational and technological innovations. Reflecting the extant variation in access to information, ideological interests, and power, a large number of such innovations—although they would have had organizational and technological forms—would have actually had ideological, political, and military functions.

In this setting, competition for power and material resources could lead to an increase in the *capacity* for achieving economies of scale, *L*, even as carrying capacity, *K_t_*, persisted at or close to prevailing population levels. This would occur as competition, exploitation, domination and resistance processes favored increasing political complexity and multiple shifting paths of economic connectedness, shaping the emergence of heterarchically and hierarchically related institutions [65], [66]. These socially structured and structuring institutions and activities would effectively consume biological well-being—that is, fitness in the context of transfer-intense, extended life history strategies (cf. ref. [20]). This would initially limit population growth. Here, political and economic factors would alter the scale and distribution of variation in biological well-being, while depressing carrying capacity, *K_t_*. Intense competition among institutions, actors, and shifting alliances would have simultaneously depended on *and* limited growth in material and labor resources. Cultural evolution may have increased the physical inputs, flow rates, and outputs (in terms of economic production and fertility), but the net demographic effect would have been near zero.

The organizational and ideological systems emerging from such cultural selection would have, in turn, increased the logistical, ideological, and technological limits on achieving economies of scale, *L*, without significantly raising population carrying capacity, *K_t_*. Only later, these cultural evolutionary processes—including diversification and competition among ideologies; economic system complexity and resilience; and proliferation of political, religious, military, and economic institutions—would have supported the development of positive feedback between population and carrying capacity.

## Conclusion

In comparative and evolutionary perspective, recent human super-exponential growth seems unlikely—or at least unfamiliar under standard theoretical models. Darwin's early insights about natural selection were crucially influenced by his reading of Malthus. In his *Notebooks on the Transmutation of Species* [67], [68], Darwin wrote (sic):

*Even a few years plenty, makes population in Men increase* & *an ordinary crop causes a dearth. take Europe on an average every species must have same number killed year with year by hawks, by cold* &*c. — even one species of hawk decreasing in number must affect instantaneously all the rest. — The final cause of all this wedging, must be to sort out proper structure,* & *adapt it to changes. — to do that for form, which Malthus shows is the final effect (by means however of volition) of this populousness on the energy of man. One may say there is a force like a hundred thousand wedges trying force every kind of adapted structure into the gaps in the oeconomy of nature, or rather forming gaps by thrusting out weaker ones.*

Here, Darwin took clear note of Malthus's insight that “geometric growth” yields startlingly short, decadal-scale human population doubling times. This underpinned Darwin's elegant argument that normal intra-decadal-scale environmental fluctuations should drive recurrent episodes of density-dependent competition within animal populations. This should limit long-term population increase but maintain conditions for what he came to call natural selection [69]. In this light, the rapid human demographic growth of the past 200 years is quite simply a remarkable population biological phenomenon. The consumption-cost model presented in this article formalizes plausible conditions in which an apparent Malthusian trap—where steady-state population regulation is theoretically expected to inhibit political-economic risk-taking, thereby limiting the adoption of technological or organizational innovations [60], [70], [71]—actually belied a very different biocultural evolutionary situation. It was not a fixed environmental carrying capacity that held historical pre-industrial populations in check. Carrying capacity and population growth alike were instead temporarily limited by intense political competition, economic dynamism, and change in the hierarchical scale and heterarchical institutional diversity of the overall cultural system. Yet, the evolution of joint political and economic institutional complexity increased the *potential* for social-network-dependent carrying capacity growth. This sets up conditions for long-term population change involving a delayed, yet extremely rapid acceleration in population increase.

**Figure 8:**
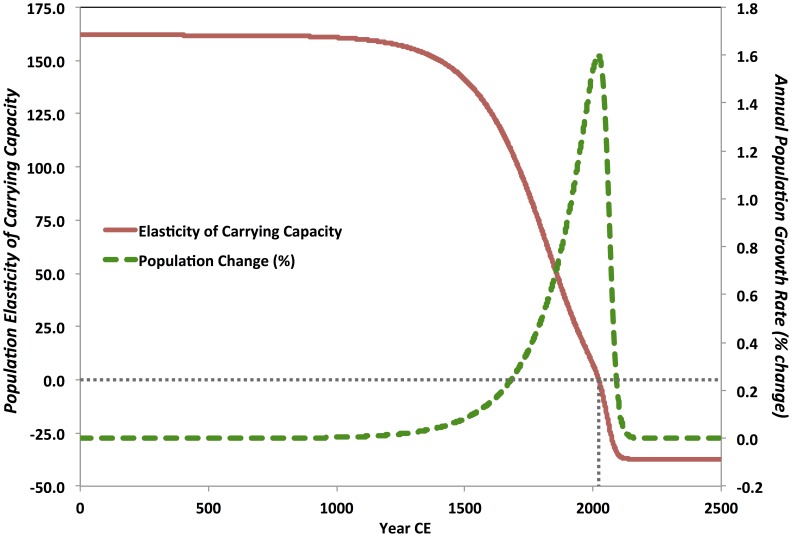
Population elasticity of carrying capacity *c_t_* (see equation 1) and % annual population change over time, for the case 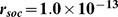; 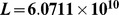; 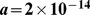; *b* = 2.20; 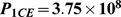; and 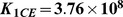. As population change reaches an equilibrium steady state, population elasticity of carrying capacity is substantially negative, revealing the equilibrium to be weak.

The consumption-cost model's success as an interpolation function, for the period 1–2012 CE, thus builds on earlier insights from the original Cohen model [22], [23]. The best-fit model population trajectories (see Table 2; Figs. 6 & 7) support the hypothesis that both modern super-exponential population increase and the incipient, likely rapid population deceleration—which we are currently beginning to face—are part of a long-term, continuous evolutionary process. Early, pre-industrial cultural changes established conditions for cumulative carrying capacity growth on centennial or millennial scales. Yet, the improved concordance of the consumption-cost model to the independent historical estimates, modern census data, *and* population projections—when compared with the standard logistic growth model and the original Cohen model—has an important implication for the future of the global human cultural system. As demographic growth continues to slow, the ecological effects of consumption will begin to have outsized negative impacts on the supply of environmental resources. Consequently, the population constituting the cultural system may be particularly precarious. As the population approaches a steady state, carrying capacity elasticity becomes negative (Fig. 8). Any positive change in population will lead to a decline in carrying capacity. Locally or regionally, this could lead to demographic-carrying-capacity system boom-and-bust cycles. Yet globally, if the costs of consumption are not reduced, an initially small decline in carrying capacity could cause recurrent fragmentation of the ideological, institutional, and technological basis for potential economies of scale, leading to long-term joint population and economic decline.

## References

[1] Malthus T (1798) An Essay on the Principle of Population. London: Printed for J.Johnson, in St. Paul's Churchyard. Available: http://www.gutenberg.org/files/4239/4239-h/4239-h.htm. Accessed 26 March 2014.

[2] Livi-Bacci M (2012) A Concise History of World Population. New York: John Wiley & Sons. 288 p.

[3] Scott JC (1977) The Moral Economy of the Peasant: Rebellion and Subsistence in Southeast Asia. New Haven: Yale University Press. 256 p.

[4] United Nations, Department of Economic and Social Affairs, Population Division (2013) World Population Prospects: The 2012 Revision, Highlights and Advance Tables. New York: The United Nations. Available: http://esa.un.org/unpd/wpp/Documentation/pdf/WPP2012_HIGHLIGHTS.pdf. Accessed 26 March 2014.

[5] Tilman D, Fargione J, Wolff B, D'Antonio C, Dobson A, et al (2001) Forecasting Agriculturally Driven Global Environmental Change. Science 292: 281–284 10.1126/science.1057544 11303102

[6] Tilman D, Cassman KG, Matson PA, Naylor R, Polasky S (2002) Agricultural sustainability and intensive production practices. Nature 418: 671–677 10.1038/nature01014 12167873

[7] Foley JA, DeFries R, Asner GP, Barford C, Bonan G, et al (2005) Global Consequences of Land Use. Science 309: 570–574 10.1126/science.1111772 16040698

[8] Godfray HCJ, Beddington JR, Crute IR, Haddad L, Lawrence D, et al (2010) Food Security: The Challenge of Feeding 9 Billion People. Science 327: 812–818 10.1126/science.1185383 20110467

[9] Poppy GM, Chiotha S, Eigenbrod F, Harvey CA, Honzák M, et al (2014) Food security in a perfect storm: using the ecosystem services framework to increase understanding. Philos Trans R Soc B Biol Sci 369: 20120288 10.1098/rstb.2012.0288 PMC3928891 24535394

[10] Galor O, Moav O (2004) From Physical to Human Capital Accumulation: Inequality and the Process of Development. Rev Econ Stud 71: 1001–1026 10.1111/0034-6527.00312

[11] Galor O, Weil DN (1998) Population, Technology, and Growth: From the Malthusian Regime to the Demographic Transition and Beyond. Working Paper. National Bureau of Economic Research. Available: http://www.nber.org/papers/w6811. Accessed 25 March 2014.

[12] Galor O, Moav O, Vollrath D (2009) Inequality in Landownership, the Emergence of Human-Capital Promoting Institutions, and the Great Divergence. Rev Econ Stud 76: 143–179 10.1111/j.1467-937X.2008.00506.x 23946551 PMC3740999

[13] Jones CI (2001) Was an Industrial Revolution Inevitable? Economic Growth Over the Very Long Run. Adv Macroecon 1. Available: http://www.degruyter.com/view/j/bejm.2001.1.2/bejm.2001.1.2.1028/bejm.2001.1.2.1028.xml. Accessed 25 March 2014.

[14] Low BS, Clarke AL, Lockridge KA (1991) Family Patterns in Nineteenth-century Sweden: Variation in Time and Space. Umeå: Demographic Data Base. 158 p.

[15] Ferguson N (2008) The Ascent of Money: A Financial History of the World. New York: Penguin. 372 p.

[16] Foucault M (1967) Madness and Civilization: A History of Insanity in the Age of Reason. New York: Routledge. 255 p.

[17] Foucault M (1977) Discipline and Punish: The Birth of the Prison. New York: Random House LLC. 346 p.

[18] Caldwell JC (1976) Toward A Restatement of Demographic Transition Theory. Popul Dev Rev 2: 321–366 10.2307/1971615

[19] Caldwell JC (2006) Demographic Transition Theory. Dordrecht: Springer. 411 p.

[20] Wood JW (1998) A theory of preindustrial population dynamics. Demography economy and well-being in Malthusian systems. Curr Anthropol 39. Available: http://www.popline.org/node/280977. Accessed 26 March 2014.

[21] Johnson-Hanks J (2008) Demographic Transitions and Modernity. Annu Rev Anthropol 37: 301–315 10.1146/annurev.anthro.37.081407.085138

[22] Cohen JE (1995) Population growth and earth's human carrying capacity. Science 269: 341–346 10.1126/science.7618100 7618100

[23] Cohen JE (1995) How Many People Can the Earth Support?New York: W. W. Norton & Company. 548 p.

[24] US Census Bureau DIS (n.d.) International Programs, World Population. Available: https://www.census.gov/population/international/data/worldpop/table_history.php. Accessed 22 February 2014.

[25] Price D (1999) Carrying capacity reconsidered. Popul Environ J Interdiscip Stud Volume 21: 5–26.

[26] Pianka ER (2011) Evolutionary Ecology, Seventh Edition. Published by the author as an e-book: Eric R. Pianka. 528 p.

[27] Odling-Smee FJ, Feldman MW, Laland KN (2003) Niche construction: the neglected process in evolution. Princeton: Princeton University Press.

[28] Ellison PT (2001) On Fertile Ground: A Natural History of Human Reproduction. Cambridge, MA, USA: Harvard University Press. 370 p.

[29] Dunsworth HM, Warrener AG, Deacon T, Ellison PT, Pontzer H (2012) Metabolic hypothesis for human altriciality. Proc Natl Acad Sci 109: 15212–15216 10.1073/pnas.1205282109 22932870 PMC3458333

[30] Lovejoy CO (1981) The Origin of Man. Science 211: 341–350 10.1126/science.211.4480.341 17748254

[31] Bentley GR, Jasienska G, Goldberg T (1993) Is the Fertility of Agriculturalists Higher Than That of Nonagriculturalists? Curr Anthropol 34: 778–785.

[32] Campbell KL, Wood JW (1988) Fertility in traditional societies. In: Diggory P, Teper S, editors. Natural Human Fertility: Social and Biological Determinants. London: Macmillan. pp.39–69.

[33] Hill KR, Hurtado AM (1996) Aché Life History: The Ecology and Demography of a Foraging People. New York: Aldine de Gruyter.

[34] Verhulst PF (1845) Recherches mathématiques sur la loi d'accroissement de la population. Nouv Mém Académie R Sci B-lett Brux 18: 14–54.

[35] Moberg A, Sonechkin DM, Holmgren K, Datsenko NM, Karlén W (2005) Highly variable Northern Hemisphere temperatures reconstructed from low- and high-resolution proxy data. Nature 433: 613–617 10.1038/nature03265 15703742

[36] Fagan BM (2000) The Little Ice Age: How Climate Made History, 1300–1850. New York: Basic Books. 272 p.

[37] Stutz AJ (2009) The “Nature of Transitions” in the Stone Age: a Comparative Perspective. In: Shea JJ, Lieberman DE, editors. Transitions in Prehistory: Papers in Honor of Ofer Bar-Yosef. Cambridge, MA, USA: Harvard University American School of Prehistoric Research. pp.477–498.

[38] Flannery KV, Marcus J (2012) The Creation of Inequality: how our prehistoric ancestors set the stage for monarchy, slavery, and empire. Cambridge, MA, USA: Harvard University Press. 622 p.

[39] Marx K (1973) Grundrisse: Foundations of the Critique of Political Economy. London: Penguin UK. 1641 p.

[40] Graeber D (2011) The divine kingship of the Shilluk: On violence, utopia, and the human condition, or, elements for an archaeology of sovereignty. HAU J Ethnogr Theory 1: 1–62.

[41] Kaplan HS, Hooper PL, Gurven M (2009) The evolutionary and ecological roots of human social organization. Philos Trans R Soc B Biol Sci 364: 3289–3299 10.1098/rstb.2009.0115 PMC2781874 19805435

[42] Kaplan HS, Robson AJ (2002) The emergence of humans: The coevolution of intelligence and longevity with intergenerational transfers. Proc Natl Acad Sci 99: 10221–10226 10.1073/pnas.152502899 12122210 PMC126651

[43] Kaplan H, Lancaster J, Robson A (2003) Embodied capital and the evolutionary economics of the human life span. Popul Dev Rev 29: 152–182.

[44] Kaplan HS, Robson AJ (2009) We age because we grow. Proc R Soc B Biol Sci 276: 1837–1844 10.1098/rspb.2008.1831 PMC2674492 19324786

[45] Cyrus Chu CY, Lee RD (2006) The co-evolution of intergenerational transfers and longevity: An optimal life history approach. Theor Popul Biol 69: 193–201 10.1016/j.tpb.2005.11.004 16406044 PMC1513193

[46] Lee RD (2003) Rethinking the evolutionary theory of aging: Transfers, not births, shape senescence in social species. Proc Natl Acad Sci 100: 9637–9642 10.1073/pnas.1530303100 12878733 PMC170970

[47] Lee R (2008) Sociality, selection, and survival: Simulated evolution of mortality with intergenerational transfers and food sharing. Proc Natl Acad Sci 105: 7124–7128 10.1073/pnas.0710234105 18458325 PMC2438215

[48] Bourke AFG (2007) Kin Selection and the Evolutionary Theory of Aging. Annu Rev Ecol Evol Syst 38: 103–128 10.1146/annurev.ecolsys.38.091206.095528

[49] Palla G, Barabási A-L, Vicsek T (2007) Quantifying social group evolution. Nature 446: 664–667 10.1038/nature05670 17410175

[50] Hamilton MJ, Milne BT, Walker RS, Burger O, Brown JH (2007) The complex structure of hunter–gatherer social networks. Proc R Soc B Biol Sci 274: 2195–2203 10.1098/rspb.2007.0564 PMC2706200 17609186

[51] Hamilton MJ, Milne BT, Walker RS, Brown JH (2007) Nonlinear scaling of space use in human hunter–gatherers. Proc Natl Acad Sci 104: 4765–4769 10.1073/pnas.0611197104 17360598 PMC1810510

[52] Hamilton MJ, Burger O, DeLong JP, Walker RS, Moses ME, et al (2009) Population stability, cooperation, and the invasibility of the human species. Proc Natl Acad Sci 106: 12255–12260 10.1073/pnas.0905708106 19592508 PMC2718330

[53] Grove M, Pearce E, Dunbar RIM (2012) Fission-fusion and the evolution of hominin social systems. J Hum Evol 62: 191–200 10.1016/j.jhevol.2011.10.012 22197359

[54] Bengtsson T, Saito O (2000) Population and Economy: From Hunger to Modern Economic Growth: From Hunger to Modern Economic Growth. New York: Oxford University Press. 514 p.

[55] Lee R, Mason A (2010) Fertility, Human Capital, and Economic Growth over the Demographic Transition. Eur J Popul Rev Eur Démographie 26: 159–182 10.1007/s10680-009-9186-x PMC2860101 20495605

[56] Lee RD (2007) Demographic Change, Welfare, and Intergenerational Transfers: A Global Overview. Ages, Generations and the Social Contract. Springer Netherlands.pp.17–43. Available: http://link.springer.com/chapter/10.1007/978-1-4020-5973-5_1. Accessed 26 March 2014.

[57] Lee RD (1986) Malthus and Boserup: A Dynamic Synthesis. The State of Population Theory: Forward from Malthus. London: Basil Blackwell. pp.96–130.

[58] Lee CT, Tuljapurkar S (2008) Population and prehistory I: Food-dependent population growth in constant environments. Theor Popul Biol 73: 473–482 10.1016/j.tpb.2008.03.001 18439637

[59] Lee CT, Puleston CO, Tuljapurkar S (2009) Population and prehistory III: Food-dependent demography in variable environments. Theor Popul Biol 76: 179–188 10.1016/j.tpb.2009.06.003 19540865

[60] Puleston C, Tuljapurkar S, Winterhalder B (2014) The Invisible Cliff: Abrupt Imposition of Malthusian Equilibrium in a Natural-Fertility, Agrarian Society. PLoS ONE 9: e87541 10.1371/journal.pone.0087541 24498131 PMC3909123

[61] Puleston CO, Tuljapurkar S (2008) Population and prehistory II: Space-limited human populations in constant environments. Theor Popul Biol 74: 147–160 10.1016/j.tpb.2008.05.007 18598711 PMC2825579

[62] Johnson AW, Earle TK (2000) The Evolution of Human Societies: From Foraging Group to Agrarian State. 2nd ed. Stanford: Stanford University Press. 384 p.

[63] Earle TK (1997) How Chiefs Come to Power: The Political Economy in Prehistory. Stanford University Press. 276 p.

[64] Kirch PV (2010) How Chiefs Became Kings: Divine Kingship and the Rise of Archaic States in Ancient Hawai'i. Berkeley: University of California Press. 286 p.

[65] Crumley CL (1995) Heterarchy and the Analysis of Complex Societies. Archeol Pap Am Anthropol Assoc 6: 1–5 10.1525/ap3a.1995.6.1.1

[66] Crumley CL (2007) Historical ecology: integrated thinking at multiple temporal and spatial scales. In: Hornborg A, Crumley CL, editors. The World System and the Earth System: Global Socioenvironmental Change and Sustainability Since the Neolithic. Walnut Creek, CA, USA: Left Coast Press. pp.15–28.

[67] Darwin CR (1838) Notebook D: [Transmutation of species (7-10.1838)]. CUL-DAR123. Available: http://darwin-online.org.uk/content/contentblock?itemID=CUL-DAR123.-&basepage=1&hitpage=113&viewtype=side#. Accessed 26 March 2014.

[68] De Beer G, Rowlands MJ, Skramovsky BM, editors (1967) Darwin's notebooks on transmutation of species. Part VI. Pages excised by Darwin. London. Available: http://darwin-online.org.uk/content/frameset?pageseq=36&itemID=F1574f&viewtype=side. Accessed 26 March 2014. 11616718

[69] Darwin CR (1859) On the origin of species by means of natural selection, or the preservation of favoured races in the struggle for life. 1st ed. London: John Murray. Available: http://darwin-online.org.uk/content/frameset?itemID=F373&viewtype=text&pageseq=1. Accessed 26 March 2014.

[70] Fitzhugh B (2001) Risk and Invention in Human Technological Evolution. J Anthropol Archaeol 20: 125–167 10.1006/jaar.2001.0380

[71] Lee RD (1988) Induced population growth and induced technological progress: Their interaction in the accelerating stage. Math Popul Stud 1: 265–288 10.1080/08898488809525278 12281209

[72] Biraben JN (1980) An essay concerning mankind's demographic evolution. J Hum Evol 9: 655–663 10.1016/0047-2484(80)90099-8

[73] Haub C (2002) How Many People Have Ever Lived on Earth? Popul Ref Bur. Available: http://www.prb.org/Publications/Articles/2002/HowManyPeopleHaveEverLivedonEarth.aspx. Accessed 11 April 2014.

[74] McEvedy C, Jones R (1978) Atlas of world population history. New York: Penguin. 378 p.

[75] Clark C (1977) Population Growth and Land Use. New York: Macmillan. 415 p.

[76] United Nations Population Division (1999) The World at Six Billion. New York: Population Division, Department of Economic and Social Affairs, United Nations Secretariat. 63 p. Available: http://www.un.org/esa/population/publications/sixbillion/sixbilpart1.pdf. Accessed 11 April 2014.

[77] Kremer M (1993) Population Growth and Technological Change: One Million B.C. to 1990. Q J Econ 108: 681–716 10.2307/2118405

[78] Blaxter SKL (1986) People, Food and Resources. New York: Cambridge University Press. 132 p.

